# EXPERIENCES IN CREATING A WRITING GROUP

**DOI:** 10.1093/geroni/igz038.851

**Published:** 2019-11-08

**Authors:** Renee J Flores, Nahid J Rianon

**Affiliations:** UT Houston McGovern Medical School, Houston, Texas, United States

## Abstract

Writing and publication in an academic setting is vital for advancing careers and knowledge. Attempting to increase scholarly productivity, our division created a physician-writing group, led by a prolific humanities expert to hone our writing skills. An unexpected outcome was realized. Using a mix of reflective, intent-driven, impromptu writing exercises and group sharing we discovered new opportunities for personal and professional growth through empathy. During these 1-hour sessions, several organic themes emerged. These included gaining greater inner-personal insight and recognizing inter-personal similarities in career paths and provider benevolence as motivation to continue when experiencing emotional fatigue and burnout. Ultimately, while honing our professional writing skills we also stimulated compassion to ourselves and our colleagues, opening new sources of resilience. We plan to continue these sessions exploring the potential multifaceted impacts on professional/academic growth these sorts of writing groups can have for geriatric and palliative medicine professionals and other healthcare providers.

